# Periodontal status and quality of life in pregnant women with both overweight/obesity and hypertension: A cross-sectional study

**DOI:** 10.4317/jced.58789

**Published:** 2021-11-01

**Authors:** Gerson-Aparecido Foratori-Junior, Bruno-Gualtieri Jesuino, Ana-Virgínia-Santana-Sampaio Castilho, Silvia-Helena-de Carvalho Sales-Peres

**Affiliations:** 1 Department of Pediatric Dentistry, Orthodontics and Public Health, Bauru School of Dentistry, University of São Paulo, Bauru, São Paulo, Brazil

## Abstract

**Background:**

The aim of this study was to assess the periodontal condition and quality of life of pregnant women affected with both overweight/obesity and arterial hypertension.

**Material and Methods:**

Pregnant women were dived into three groups: with overweight/obesity and hypertension (G1 = 23), with overweight/obesity without hypertension (G2 = 31) and with normal BMI and without hypertension (G3 = 38). They were evaluated regarding: contextual variables (age, socioeconomic level and anthropometric parameters); periodontal status; and quality of life (adapted version of Oral Health Impact Profile – OHIP-14). ANOVA, Kruskal-Wallis, chi-square and binary logistic regression model were adopted (*p*< 0.05).

**Results:**

There were no intergroup differences for age (*p* = 0.700), education level (*p* = 0.119) and gestational weight gain (*p* = 0.415), nevertheless G2 differed from G3 regarding household monthly income (*p* = 0.040). G2 had higher prevalence of bleeding on probing than G3 (*p* = 0.001), but G1 and G2 presented higher prevalence of periodontitis (*p*< 0.001). Household monthly income (adjusted OR = 0.71; 95% CI = 0.52 - 0.98; *p* = 0.038) and hypertension (adjusted OR = 3.70; 95% CI = 1.16 - 11.80; *p* = 0.026) remained in the final logistic regression model [X2(4) = 21.79; *p* = 0.0002; R2 of Nagelkerke = 0.284]. G1 showed worse impact on quality of life, mainly regarding physical pain (*p*< 0 .001), psychological discomfort (*p*< 0 .001), physical disability (*p*< 0 .001), social disability (*p* = 0.005) and handicap (*p*< 0 .001).

**Conclusions:**

In conclusion, maternal excessive weight is associated with periodontitis during pregnancy. Moreover, the presence of hypertension in overweight pregnant women seems to be determinant to negatively influence their quality of life, resulting in physical, psychological and social damages.

** Key words:**Hypertension, obesity, overweight, periodontitis, quality of life.

## Introduction

Obesity is a chronic inflammatory disease that has affected several countries around the world. Based on measured body weight and height data from 128.9 million children, adolescents and adults from all countries in the world, obesity prevalence increased in every country between 1975 and 2016 (1). Obesity is associated with several comorbidities such as type 2 diabetes mellitus, cardiovascular diseases (hypertension, myocardial infarction and stroke), depression and some types of cancer. Also, obesity might lead to reduced quality of life, unemployment, lower productivity and social disadvantages (2).

Hypertension is also a global public health challenge. Obesity, high dietary sodium intake, low dietary potassium intake, alcohol consumption, lack of physical activity and unhealthy diet are risk factors-related lifestyle for hypertension (3). Previous studies have highlighted the association between both obesity and hypertension with periodontitis (4-8). The biological mechanism that explains this association refers to the generalized inflammatory state of the body of individuals with obesity and hypertension due to the high levels of inflammatory mediators, such as pro-inflammatory cytokines (IL-1, IL-6, IL-8 and TNF-a), adipokines (leptin, adiponectin, resistin and inhibitors of plasminogen activator-1) and other bioactive substances, such as reactive oxygen species, by adipose tissue (6,7).

During pregnancy there are several changes in the body of women, mainly due to high levels of estrogen and progesterone and, consequently, the impact on the immune response. Recent studies have found an association between periodontitis and overweight/obesity during pregnancy (9-16). In addition, some of them pointed out a high prevalence of arterial hypertension during pregnancy in women with overweight/obesity (11-16). Based on the authors’ knowledge, no study has evaluated the prevalence of periodontitis in pregnant women affected by both obesity and arterial hypertension.

Considering the high prevalence of comorbidities to which pregnant women are currently exposed and their damages in oral condition, the aim of this study was to assess the periodontal condition and quality of life of pregnant women affected with both overweight/obesity and arterial hypertension.

## Material and Methods

This observational and cross-sectional study followed the Strengthening the Reporting of Observational Studies in Epidemiology (STROBE) guidelines (17).

-Ethical approval

The Declaration of Helsinki (1964), and its subsequent amendments, were respected. Also, this study was approved by the Research Ethics Committee from Bauru School of Dentistry, University of São Paulo (CAAE 06624519.3.0000.5417) and all patients provided written consent before their participation.

-Sample description

Initially, 103 pregnant women were consecutively recruited between November/2020 to March/2021 from public health sector in Bauru, São Paulo, Brazil. The inclusion criteria in the sample were: age between 20-35 years old; regularly enrolled in the public health service; regular follow-up during medical prenatal care; being during the 3rd trimester of pregnancy (between 27-39th gestational week) and adequate neurological and motor condition. The following exclusion criteria were adopted: smokers; alcohol and/or illicit drugs users; twin pregnancy; pregnant women who required absolute rest; with underweight (BMI < 18.5 kg/m2); with suspected or confirmed SARS-CoV-2 infection (self-reported information); with diabetes mellitus before pregnancy or gestational diabetes mellitus diagnosis; individuals under orthodontic treatment; who had previous history of surgical treatment for periodontitis (self-reported information); who were using drugs that could harm the periodontal tissue (e.g., immunosuppressive, anticonvulsant or calcium channel-blocking drugs, such as cyclosporine, phenytoin or nifedipine, respectively); and who had lost more than two teeth per hemiarch.

Eleven participants were excluded from the sample due to: had mild symptoms of COVID-19 during the second trimester of pregnancy without confirmation of diagnosis (n = 4); gestational diabetes mellitus diagnosis (n = 3); had confirmation of SARS-CoV-2 infection (n = 2); multiple tooth loss (n = 1); smokers (n = 1). Therefore, the sample was composed by 92 pregnant women divided into: with overweight/obesity and hypertension (G1 = 23); with overweight/obesity without hypertension (G2 = 31); and with normal BMI and no hypertension (G3 = 38). Overweight/obesity and normal BMI were defined as pre-pregnancy BMI equals to or higher than 25 kg/m2 and BMI between 18.50 to 24.99 kg/m2, respectively (18). Hypertension was considered when women had systolic blood pressure (SBP) equals to or higher than 140 mmHg and/or diastolic blood pressure (DBP) equals to or higher than 90 mmHg (19). Patients’ pre-gestational weight was obtained from the prenatal medical files and their height was measured by a calibrated stadiometer (Wood 2.20; WCS Ind., Curitiba, Paraná, Brazil). Blood pressure was measured by a calibrated digital sphygmomanometer (G-Tech; Accumed Produtos Médico Hospitalares Ind., Duque de Caxias, Rio de Janeiro, Brazil). In addition to the pre-gestational weight collected from medical files, the weight of patients in the third trimester of pregnancy was obtained using a calibrated automatic scale (MIC model 300PP, maximum capacity 300 kg; Micheletti Ind., São Paulo, São Paulo, Brazil).

The sample was also evaluated according to contextual variables such as age, educational level and household monthly income. Education level was graded on a numeric scale from illiterate to doctorate. These levels were previous described by Foratori-Junior *et al*. (14). Similarly, household monthly income was categorized in levels also described by Foratori-Junior *et al*. (14).

-Periodontal examination

Self-reported information regarding daily tooth brushing, daily flossing during pregnancy and regular dental care by professional were obtained from patients. For each patient, the percentage of dental surfaces (buccal or palatal/lingual surfaces) with visible dental biofilm was recorded.

Full-mouth periodontal examination (six sites per tooth - mesial, central and distal, both in the buccal and palatal/lingual surfaces) was performed by a calibrated dentist (kappa = 0.92) with a manual North Carolina-type periodontal probe (QD.320.05; Quinelato, Schobell Ind. Ltda, Rio Claro, São Paulo, Brazil), excluding the third molars. Periodontitis was considered if (i) interdental clinical attachment loss (CAL) was observed at two or more non-adjacent teeth, or buccal or oral CAL of ≥ 3 mm with probing pocket depth (PPD) of > 3 mm at two or more teeth (20). Subsequently, patients diagnosed with periodontitis were classified as stages I, II, III, and IV of the disease, according to Tonetti *et al*. (20). The prevalence of sites which showed bleeding on probing (BOP) after 10 seconds was registered in percentage (21). Those patients without periodontitis were classified as healthy (without signs of gingivitis - ≤ 10% sites with BOP); localized gingivitis (10-30% of sites with BOP) and generalized gingivitis (≥ 30% of sites with BOP) (22).

-Quality of Life assessment 

The short version of the Oral Health Impact Profile questionnaire (OHIP-14) was used, which was applied through a standardized interview to avoid different interpretations among patients, minimizing the subjectivity of the questionnaire. The following dimensions of OHIP-14 were accessed: functional limitation, physical pain, psychological discomfort, physical disability, psychological disability, social disability, and handicap (23). The total OHIP-14 score was obtained and, furthermore, the patients were categorized into: “without impact of oral condition on quality of life” (OHIP-14 score equal to zero); 0 < OHIP-14 ≤ 9 was classified as “low impact”; 9 < OHIP-14 ≤ 18 was classified as “moderate impact”; and 18 < OHIP-14 ≤ 28 was classified as “high impact” (13,16).

-Statistical analysis 

Data were analyzed using the IBM Statistical Package for the Social Sciences (SPSS) software version 25 (IBM SPSS Statistics for Windows, Version 25.0. released 2017; IBM Corp., Armonk, NY, USA). According previous studies (12,13,15) for sample size, the Hosmer and Lemeshow protocol for logistic regression analysis was considered, which allows the inclusion of 15 cases for each combination of independent variables. In this study, dichotomization of periodontitis (0, no periodontitis; 1, periodontitis) was performed for binary logistical regression.

Initially, the variables were tested for normality using the Kolmogorov-Smirnov test. For quantitative variables with a normal distribution, the analysis of variance (ANOVA) was used with Tukey as post-hoc test for multiple comparisons. The Kruskal-Wallis test was used for quantitative variables without a normal distribution and qualitative ordinal, with Dunn as post-hoc test for multiple comparisons. For the nominal qualitative variables, the Chi-square was used. Binary logistic regression (stepwise backward - likelihood ratio) was performed for analyze which independent variables were associated with outcome (presence of periodontitis). Hosmer-Lemeshow, collinearity, and residual analyses were implemented to explain the results obtained through logistic regression. A significance level of 5% was adopted.

## Results

Table 1 shows the comparison between groups for contextual and periodontal variables. The average age of the sample was 28.50 years old (± 5.2). There were no differences between groups with regard to educational level. However, more than 65% and 80% of G1 and G2, respectively, pointed out that the family receives 3 or less minimum wages monthly. In contrast, more than 60% of the G3 declared that their family receives 3 or more minimum wages monthly (*p* = 0.040).

**Table 1:**
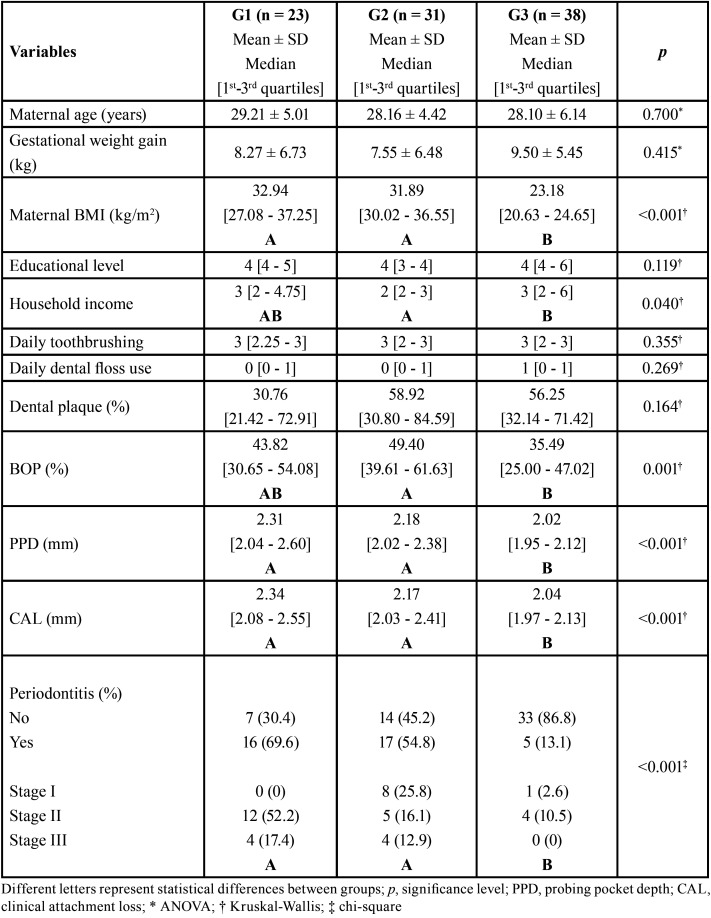
Comparison of contextual and periodontal variables between groups.

The groups did not differ in terms of oral hygiene habits. Consequently, they showed no differences in the prevalence of dental surfaces with visible dental biofilm (*p* = 0.164). Nevertheless, G2 presented higher prevalence of sites with BOP when compared to G3 (*p* = 0.001). G1 and G2 showed higher PPD and CAL compared to controls, but without differences between them. Almost 69% (n = 16), 55% (n = 17) and 13% (n = 5) of G1, G2 and G3, respectively, had periodontitis during pregnancy (*p* < 0.0001). Approximately 26% (n = 8) and 2.5% (n = 1) of G2 and G3, respectively, presented periodontitis in stage I. 52% (n = 12), 16% (n = 5) and 10% (n = 4) of G1, G2 and G3, respectively, presented stage II of periodontitis. 17% (n = 4) and 13% (n = 4) of G1 and G2, respectively, had stage III of periodontitis. No patient had stage IV of periodontitis. 4% (n = 1), 8% (n = 2) and 17% (n = 4) patients from G1 were classified as healthy, localized and generalized gingivitis, respectively. 9% (n = 3) and 35% (n = 11) of G2 were diagnosed with localized and generalized gingivitis, respectively. 7% (n = 3), 26% (n = 10) and 52% (n = 20) of G3 were classified as healthy, localized and generalized gingivitis, respectively (Table 1).

In order to understand which independent variables are associated with periodontitis during pregnancy, we performed a binary logistic regression (Table 2). The following variables were inserted in the initial model: maternal age, household monthly income, maternal BMI and presence of hypertension. In the multicollinearity analysis, all independent variables presented tolerance values of >0.70 and variance inflation factor values of < 2. The final model was significant [X2(4) = 21.79; *p* = 0.0002; R2 of Nagelkerke = 0.284] and was composed by all variables inserted in the initial model. However, presence of hypertension (adjusted OR = 3.70; 95% CI = 1.16 - 11.80; *p* = 0.026) and household monthly income (adjusted OR = 0.71; 95% CI = 0.52 - 0.98; *p* = 0.038) were associated with periodontitis. The overall accuracy of the final model was 73.9%. In the Hosmer–Lemeshow analysis, a chi-square value for the final model of 9.21 for 8 degrees of freedom (*p* = 0.324) was obtained.

**Table 2:**
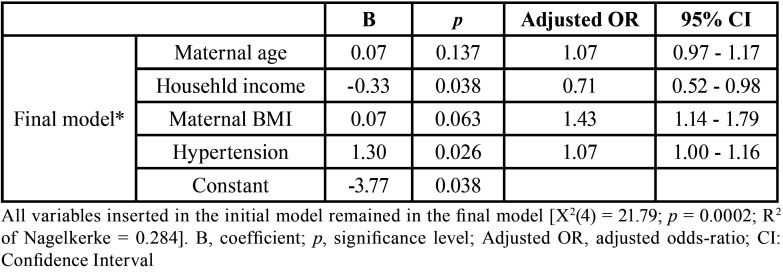
Binary logistic regression model showing the independent variables related to periodontitis during the third trimester of pregnancy.

Pregnant women with overweight/obesity and hypertension presented higher impact of oral health on quality of life, showing worst parameters related to physical pain (*p* = 0.0004), psychological discomfort (*p* = 0.0001), physical disability (*p* < 0.0001) and handicap (*p* = 0.0007) when compared to G2 and G3. G1 also had worst functional limitation (*p* = 0.0056) and psychological disability (*p* = 0.0049) when compared to G3 and worst social disability (*p* = 0.0059) when compared to G2. Almost 48% of G1 were classified as moderate or strong impact of oral health on quality of life, whereas approximately 74% and 60% of G2 and G3, respectively, had low impact on quality of life (Table 3).

**Table 3:**
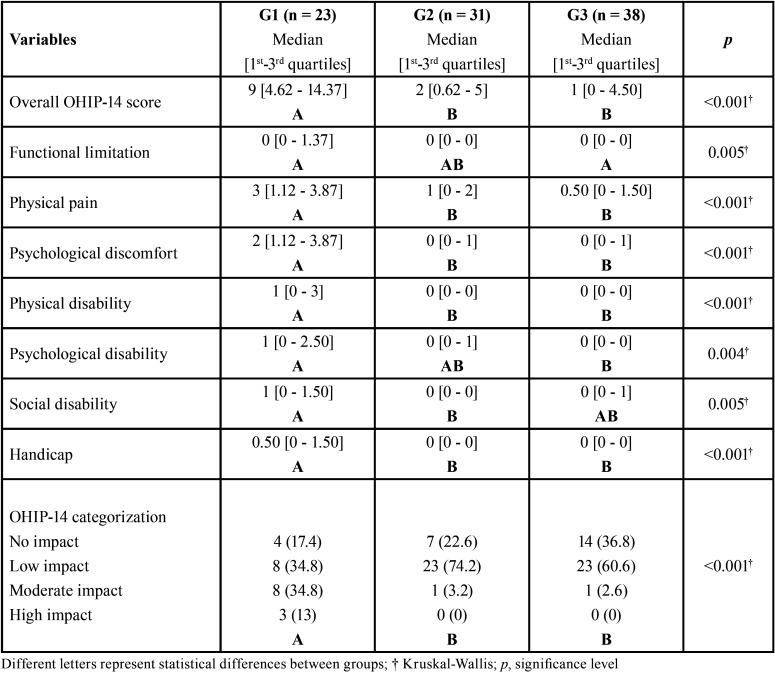
Comparison of dimensions and overall score of OHIP-14 between groups.

## Discussion

This study demonstrates that maternal excessive weight is associated with periodontitis during pregnancy. Yet, although there is no difference in respect of periodontal parameters between groups of obese women with and without hypertension, the presence of hypertension in overweight pregnant women seems to be determinant to negatively influence their quality of life, in respect of physical, psychological and social dimensions.

Some studies have already reported the association of low socioeconomic status with periodontitis (14,15,24), and also with overweight (25). In this study, G1 and G2 showed higher PPD and CAL compared to controls, and although there were no differences between groups with regard to educational level, more than 65% and 80% of G1 and G2, respectively, pointed out that the family receives only 3 or less minimum wages monthly. This situation may be explained by the fact that patients who have a lower household monthly income use to eat cheaper foods, which in most cases are more caloric and less nutritious, contributing to a higher BMI (14,15,24). Likewise, pregnant women with lower monthly income have less access to health care or oral health prevention programs (15), contributing for a worse periodontal status.

In this study, the groups did not differ in terms of oral hygiene habits and, consequently, they showed no differences in the prevalence of dental surfaces with visible dental biofilm (*p* = 0.164). Therefore, the hypothesis that best explain the higher prevalence of periodontitis in G1 and G2 (*p* < 0.001) is related to patients’ systemic impairments (Table 1). Maternal excessive weight can lead to an inflammatory condition in the patient’s body, as the intake of macronutrients is associated with the accumulation of lipids in adipocytes and the expansion of this adipose tissue, which, in turn, may initiate an inflammatory process through the production of cytokines pro-inflammatory, such as TNF-α, IL-6 and C-reactive protein (CRP) (10,24,26). Thus, even with a small amount of dental plaque, pregnant women with overweight/obesity may present an exacerbated periodontal inflammation (24,26).

In a systematic review and meta-analysis, Munoz-Aguilera *et al*. revealed that periodontitis is associated with hypertension; however, there is no clear evidence yet about the cause-effect relationship of these outcomes (7). Clinical and experimental evidence suggest that this direction of the association could be mediated through hypertension causing microcirculatory changes in of the gingival tissue leading to ischemia, increased inflammation, and/or altered microbial composition of the dental biofilm (7,27-29). Recently, morbidly obese patients with hypertension had worst periodontal parameters than normotensive morbidly obese patients (8). Also, previous studies showed obese/overweight pregnant women had higher prevalence of both hypertension and periodontitis during the third trimester of pregnancy (12-16). In this study, there were no differences for periodontal parameters between hypertensive and normotensive pregnant women with overweight (Table 1), but besides the household monthly income, maternal hypertension was associated with periodontitis in the final model of binary logistic regression (adjusted OR 1.07 95% CI 1.00-1.16, *p* = 0.026; Table 2).

OHIP-14 has been used to investigate the impact oral health on quality of life. However, it is important to emphasize that quality of life is a subjective dimension in which several health determinants, in addition to those related to oral health, can influence (16). Caracho *et al*. showed that overweight women assisted by Brazilian public health-care system presented a higher prevalence of arterial hypertension and periodontitis, and also showed more negative impact on quality of life, mainly related to physical and psychological dimensions (13). Similarly, Foratori-Junior *et al*. showed that pregnant women with obesity presented higher prevalence of arterial hypertension and periodontitis and also poorer quality of life than eutrophic pregnant women, mainly in respect of functional limitation, physical disability and handicap (16). In this study, pregnant women with both overweight/obesity and hypertension presented more negative impact on quality of life, resulting in physical, physiological and social damages (Table 3). Thus, our findings suggest that even without difference for periodontal parameters between hypertensive and normotensive pregnant women with overweight, when hypertension is associated with overweight they seem to be detrimental to the physical, psychological and social dimensions of the patients’ quality of life.

This study has some limitations. Future cohort population-based studies could be better to understand the association between outcomes and their cause-effect relationships. This study grouped obese and overweight patients together, because it could be difficult to recruit pregnant women with hypertension only with obesity or only with overweight. In addition, biological tests are necessary to better understand the biological mechanisms in respect of the association of periodontitis and systemic disorders. Although the limitations, this study is important to understand the association between maternal BMI, hypertension, periodontitis and quality of life of pregnant women. This understanding is necessary for clinical dentists to adopt interdisciplinary protocols for the care of women during pregnancy in order to contribute to adequate women’s oral health and general health for them and their babies.

In conclusion, our findings suggest that besides maternal excessive weight is associated with periodontitis during pregnancy, the presence of hypertension in overweight pregnant women seems to be determinant to negatively influence their quality of life, resulting in physical, psychological and social damages.
